# Where to Sit? Type of Sitting Matters for the Framingham Cardiovascular Risk Score

**DOI:** 10.3934/publichealth.2016.3.577

**Published:** 2016-08-15

**Authors:** Heini Wennman, Tommi Vasankari, Katja Borodulin

**Affiliations:** 1 National Institute for Health and Welfare, PO Box 30, FI-00271 Helsinki, Finland; 2 UKK-Institute for Research and Health Promotion, PO Box 30, FI-33501 Tampere, Finland

**Keywords:** sedentary behavior, type-specific sitting, the Framingham risk score, cardiovascular health

## Abstract

**Background:**

Current evidence on associations of type-specific sedentary behavior with cardiovascular disease (CVD) is limited to mainly screen-time sedentary behavior (SB). We aimed to study the associations of type-specific and total time spent sitting with the Framingham 10-year cardiovascular disease risk score (Framingham score) in Finnish adults.

**Methods:**

Data comprise the National FINRISK 2007 and 2012 health examination surveys with 10,185 participants aged 25–74 years, apparently free of CVD. Participants reported average daily time spent sitting in different locations: work-related sitting, at home in front of television (TV), at home in front of computer, in a vehicle, and elsewhere. Total SB time was calculated from these context-specific self-reports. Accelerometer-based sedentary time was assessed in 988 FINRISK 2012 participants. Framingham score was calculated using information on blood pressure and its medication, cholesterol levels, age, diabetes status, and smoking. Analyses were adjusted for age, study year, education, employment status, leisure time physical activity, and body mass index.

**Results:**

Out of several type-specific sitting behaviors, only TV sitting showed systematic associations with the Framingham score in both genders. The lowest Framingham risk was found for TV sitting from 6 minutes to less than 1 hour daily. Of other types of sitting, computer sitting was inversely associated with the Framingham risk in men only. Total self-reported sitting time did not show significant associations with the Framingham score, but instead higher objectively assessed sedentary time showed higher Framingham risk in men.

**Conclusions:**

TV sitting showed most systematic associations with CVD risk score. This suggests that of all types of SB, reducing TV sitting should be targeted for reducing CVD risk.

## 1. Introduction

There is a growing body of evidence suggesting that long times spent sedentary may harm health [1]–[5]. The evidence has particularly been shown for prospective studies on cardiovascular diseases (CVD) [6],[7], but also cross-sectional studies on CVD risk factors [8]–[11]. The proposed mechanisms linking sedentary behavior (SB) with CVD include alterations in muscles' lipid and glucose uptake mechanisms, and also changes in vascular function [12]–[14]. There are also studies with controversial findings, suggesting less systematic associations between SB and health outcomes [15] and non-linear associations [16].

Television (TV) viewing, screen time, or total sitting time have been the most studied exposure variables in epidemiological research [17], out of which sitting by TV has been proposed to be the most deleterious [18]–[21]. Of other types of sitting, occupational sitting has been suggested to not necessarily being harmful to health [4],[22],[23] and riding a car or in a bus to show diverse associations with health [21],[24]–[26]. Using a computer, when considered separate from screen time, has shown both a direct and inverse association against health outcomes [27],[28].

It is important to understand that SB is not a single construct as correlates of SB vary significantly by the type of SB measured [29],[30]. Therefore it is recommended not only to measure total SB, but also type-specific SB that will help us to better understand the epidemiology of SB and potentially intervene in a more tailored manner [14],[30].

The Framingham Risk Score is a widely used risk calculator for individual CVD risk [31], which can be calculated using information on the classic risk factors of age, blood pressure, total and HDL-cholesterol, diabetes, smoking and use of medication. Individuals with a Framingham score of more than 20% are considered to be at high risk [31]. One previous study on SB and Framingham score has suggested higher SB to associate with a higher Framingham score [32]. Others have shown that physical activity is associated with a beneficial Framingham risk score [33] and that physical activity has an inverse association with CVD mortality, independent of the baseline Framingham risk score [34]. Furthermore, one study found a combination of unhealthy behaviors, such as low physical activity, high sitting, and poor sleep to associate with the Framingham risk score [35]. To our knowledge, the Framingham risk score has not been used to study associations with type-specific SBs.

Based on the current amount of evidence, it is too early to judge the health risks for type-specific SBs. To fill this gap, our aim was to study the association of different types of self-reported sitting, their combined total sitting, and accelerometer-based sedentary time with the predicted 10-year CVD risk in a representative sample of 10,185 adults. The types of sitting include work-related sitting, sitting at home in front of TV or computer, in a vehicle, and elsewhere.

## 2. Methods

### 2.1. Study Population

Data comprise the National FINRISK 2007 and 2012 studies that are independent, cross-sectional population based health examination surveys that aimed at monitoring chronic disease risk factors in the adult population of Finland [36]. The study protocol in both surveys follows the recommendations of the WHO MONICA Study [37] and the later recommendations of the European Health Risk monitoring project [38]. The FINRISK studies, respectively in 2007 and 2012, invited a stratified random sample of 10,000 Finnish men and women aged 25 to 74 to participate in a health examination and to answer questionnaires assessing health behaviors and health status. In 2007 the participation rate was 68% (n = 6733) and in 2012 65% (n = 6424). Ethical approval was given by the Coordinating Ethics Committee of the University Hospital District of Helsinki and Uusimaa. Participants gave a written, informed consent.

At the health examination a trained nurse measured blood pressure, weight and height and drew a blood sample. Blood pressure was measured three times, using mercury sphygmomanometers, from the right upper arm in a sitting position and an average of second and third measurements was computed. Height was measured with a stadiometer without shoes to the nearest 0.1 cm. Weight was measured in light clothing to the nearest 100 grams with a beam balance scale. Body mass index (BMI) was calculated as weight in kilograms divided by squared height in meters (kg/m^2^). Venous blood samples were taken after at least a 4-hour fast. All blood samples were analyzed in the laboratory of the National Institute for Health and Welfare (Helsinki, Finland). Serum total cholesterol, high density lipoproteins (HDL-C), and triglycerides were analyzed using enzymatic methods (Abbott Laboratories, Abbott Park, Illinois, U.S.A). The health examination protocol is described in detail elsewhere [36].

### 2.2. Measurement of the Framingham Risk Score

The Framingham score was calculated as suggested by D'Agostino et al [31]. The risk score estimates a percentage risk for total CVD within the next 10 years. The risk score is sex specific and based on information about age in years, the levels of systolic blood pressure (mmHg), total and HDL-cholesterol (mg/dl), diabetes (yes or no), smoking (yes or no) and use of blood pressure medication (yes or no). Measured information about blood pressure, serum total and HDL-cholesterol were available from the health examination. Health behaviors and use of medications were assessed on the questionnaire. Blood pressure medication was considered to be used if the person reported use within the past week. A person was considered diabetic if either having physician-diagnosed diabetes or reporting the use of diabetes medication.

### 2.3. Measurement of Sitting

Time spent sitting was self-reported in a questionnaire, where participants reported their average daily type-specific sitting times in hours and minutes: during work days in the office or equivalent (hereafter work-related sitting), at home in front of TV (hereafter TV sitting), at home in front of computer (hereafter computer sitting), in a vehicle (hereafter vehicle sitting), and elsewhere (hereafter sitting elsewhere). These questions resemble the validated Marshall sitting questionnaire [39] which has shown high correlation coefficients on reliability and validity for context-specific sitting when studied against accelerometry. The sitting questions were kept precisely the same in the 2007 and the 2012 Surveys. Type-specific sitting times were calculated and, based upon the sum of type-specific durations, a sum of total time spent sitting (hereafter total sitting) was calculated.

Educational levels of low, medium and high were based on self-reported total educational years adjusted for with birth cohort. Employment status was dichotomized into working or not working. Those not working included the retired, unemployed (without work), or homemakers. Leisure time physical activity was assessed by a question, “How much do you exercise and stress yourself physically in your leisure time?” Response options were (1) In my leisure time, I read, watch TV, and work in the household with tasks which do not make me move much and which do not physically tax me; (2) In my spare time, I walk, cycle or exercise otherwise at least 4 hours per week, excluding travel to work; (3) In my spare time, I exercise to maintain my physical condition for at least 3 hours per week; and (4) In my spare time, I regularly exercise several times a week in competitive sports or other heavy sports. The questionnaire has shown good criterion validity against morbidity and mortality [40] and moderate correlation against accelerometer counts among the working age population [41].

### 2.4. Measurements with Accelerometry

In 2012, a subsample (n = 1122) of the FINRISK Study participants wore Hookie accelerometers (Traxmeet Ltd., Espoo, Finland) at their waist during 7 consecutive days to assess physical activity and SB. The devices were worn only during waking hours and were removed during water exposure activities. The participants kept a diary for non-wear time and for physical activity and sleep.

The activity data were registered as raw acceleration data by 100 Hz sample rate with 2GB internal flash memory. Time spent in SB (including sitting and lying down) was recognized from raw acceleration data based on low intensity of movement using mean amplitude deviation and device orientation in relation to identified upright position defined at the end of each epoch [42]. Similarly, based on mean amplitude deviation and device orientation were variables describing physical activities of different intensities identified [43]. With the information on the raw data from the tri-axial measurement, body posture was detected, for example for standing, sitting, or lying down. The orientation of the accelerometer was determined from normal walking in an upright position, when gravity remained constant, and this information was used as a reference value. Against the reference value, all other body postures were calculated. With this method, standing, sitting and lying down could be separated from each other reliably [43]. For the purposes of this study, the daily average of total SB and total physical activity of at least moderate intensity were utilized. Participants with at least 10 hours during 4 days or more of accelerometer measurements were considered for the analyses (n = 988).

### 2.5. Statistical Methods

Out of those who participated (n = 13,157), we excluded persons for whom calculation of the Framingham risk score was impossible due to missing data on included variables (n = 1417). Then we excluded participants with a history of CVD (including myocardial infarction, stroke, bypass surgery, angioplasty, angina pectoris, and heart failure, n = 1001). Furthermore, those with missing information on self-reported sitting or reporting very high values of daily sitting (total sitting 24 hours or more, TV sitting 20 hours or more, or computer sitting 12 hours or more) were excluded (n = 363), as well as those with missing information on any other variables used in the statistical models (n = 191). Finally 4649 men and 5536 women (n = 10,185) were included in the analyses. Those excluded were more often men than women, they were significantly older, they had higher self-reported total sitting time, higher TV time, higher BMI and a higher Framingham score than the included (*p* < 0.001 for all).

Descriptive statistics included means and standard deviations and frequencies for categorical variables. The main statistical method was analysis of variance (ANOVA). The Framingham score was skewed and therefore a natural logarithm transformation was undertaken. Type-specific sitting times were categorized according to their respective distribution into 4 (work-related sitting, sitting elsewhere), 5 (computer sitting, vehicle sitting) or 6 (TV sitting) groups, representing meaningful groupings close to one hour time intervals. Total sitting was categorized into 5 groups, representing meaningful groupings and cutoff values. Having many categories allowed us to understand potential cut-off values in sitting and curvilinear associations for CVD risk. For accelerometer-based sedentary time, categorization was based on thirds. Results for ANOVA models are presented in F-, *p*, and *df*-values, as well as pairwise comparisons (with Bonferroni adjustment for multiple comparisons) with means presented as back transformed geometric means. All models were adjusted for age, study year, education and employment status, leisure time physical activity, and BMI. For accelerometer-based sedentary time, models were the same, except that objectively assessed moderate-to-vigorous physical activity time was used instead of self-reported leisure time physical activity and study year was not included as a covariate since all subjects with accelerometer-based SB data originated in the FINRISK 2012 cohort. Statistical analyses were conducted in IBM SPSS v.22 with statistical significance considered at *p* < 0.05.

## 3. Results

Characteristics of the study population by gender are presented in Table 1. The mean age of men was 50 years and of women 49.3 years. Participants had gone to school for an average of 14 years, and most of them were engaged in working life. The average Framingham score was 15.6% in men and 7.5% in women. Men reported daily total sitting time of 432 minutes (7 h and 12 minutes) and women of 391 minutes (6 h 31 minutes). Highest type-specific sitting values were reported for daily work-related sitting (men 210 minutes and women 229 minutes). Accelerometer-based daily mean sitting and lying was 559 minutes (9 h 19 minutes) in men and 527 minutes (8 h 47 minutes) in women.

### 3.1. Type-specific Sitting

Most systematic findings between the types of sitting and the Framingham score were found for TV sitting (F = 5.640, *df* = 5, *p* < 0.001 in men and F = 4.140, *df* = 5, *p* = 0.001 in women). In men (Table 2), both the pairwise comparisons and linear trend test showed that highest risk scores were found for those with TV sitting of 4 hours or more. The lowest Framingham score was found for TV sitting categories below 1 hour per day. Findings on TV sitting were similar among women (Table 3), with higher TV sitting suggesting a higher Framingham risk. Highest risk was observed for TV sitting from 2 to 3 hours.

Of other types of sitting, computer sitting showed a significant overall effect on the Framingham score in men (F = 4.250, *df* = 4; *p* = 0.002), but not in women (F = 1.239, *df* = 4; *p* = 0.292). The pairwise comparisons for men indicated that the highest Framingham score was found for those with no computer sitting at all, which was significantly different from computer sitting of 3 hours or more. The inverse linear trend for computer sitting was also significant in men (*p* < 0.001).

Work-related sitting, sitting in vehicle or sitting elsewhere did not show any statistically significant associations with the Framingham score in either gender.

In both men and women and for all types of sitting, the differences in the Framingham score were small across the sitting categories. For TV sitting categories, the percentage of risk varied between 8.15%–9.54% in men and 3.91%–4.28% in women. These differences, albeit statistically significant, suggest clinically modest findings.

### 3.2. Total Sitting

Self-reported total sitting showed no significant overall association with the Framingham score in either men (Table 2) or women (Table 3). In women a significant quadratic trend for self-reported total sitting time was observed (*p* = 0.034) suggesting a curvilinear, U-shaped association of total sitting with the Framingham risk. Accelerometer-based thirds of total amount of time spent sitting and lying down had a significant overall and a direct linear association with the Framingham score, but this was found in men only. Pairwise comparisons revealed a higher Framingham score in the group with 581 minutes and more time sedentary as compared to 0–499 minutes of sedentary time.

**Table 1.** Descriptive statistics of the study population.

	Men n = 4649		Women n = 5536	
	Mean (± SD)	Min-Max	Mean (± SD)	Min-Max
Age (years)	50.0 (± 13.7)	25–74	49.3 (± 13.8)	25–74
Educational years	13.0 (± 3.8)	0–33	13.8 (± 4.0)	1–50
Body Mass -index (kg/m^2^)	27.2 (± 4.1)	15.9–68.2	26.6 (± 5.3)	16.1–53.5
Framingham risk score %	15.6 (± 13.9)	0.5–98.1	7.5 (± 8.0)	0.2–79.7
Total sitting (minutes)	432.4 (± 212.7)	0.0–1380	390.7 (± 202.2)	0.0–1260
Work-related sitting (minutes)	152.7 (± 180.5)	0–900	160.4 (± 181.6)	0–1035
Work-related sitting (minutes) (only those currently active in working life)	210 (± 181.6)	0–900	228.8 (± 179.0)	0–1035
TV sitting (minutes)	133.4 (± 94.6)	0.0–1200	127.3 (± 88.2)	0.0–1200
Computer sitting (minutes)	55.4 (± 74.7)	0.0–720	40.9 (± 60.1)	0.0–720
Sitting in vehicle (minutes)	60.3 (± 91.3)	0.0–960	31.7 (± 41.5)	0.0–600
Sitting elsewhere (minutes)	30.7 (± 62.9)	0.0–720	30.4 (± 66.5)	0–900
Sitting and lying down (minutes)*	559.2 (± 110.5)	298.2–1017.9	527.4 (± 89.8)	238.5–847.7
Moderate to vigorous physical activity (minutes)*	38.5 (± 20.8)	1.7–184.6	40.7 (± 23.9)	0.8–137.2
No leisure time physical activity %	19.3 %		20 %	
Currently active in working life %	69.9 %		67.4 %	

*men n = 429, women n = 559

**Table 2.** Framingham risk score in men by type of sitting.

Sedentary measure	Framingham risk score % (Geometric mean and corresponding 95% CI)	Overall F-test, *p*-value, *df*, trend
Total sitting		0.480; *p* = 0.751; *df* = 4
< 4 hrs	9.11% (8.78–9.44)	
4–5.9 hrs	9.02% (8.72–9.31)	
6–7.9 hrs	9.06% (8.74–9.39)	
8–9.9 hrs	9.15% (8.81–9.50)	
10– hrs	8.92% (8.62–9.22)	
Sitting in vehicle		1.438; *p* = 0.219; *df* = 4
0 hrs	8.92% (8.63–9.23)	
0.1–0.9 hrs	8.97% (8.73–9.23)	
1–1.9 hrs	9.14% (8.86–9.43)	
2–2.9 hrs	9.47% (9.00–9.95)	
3– hrs	9.16% (8.65–9.69)	
TV sitting		5.640; *p* < 0.001; *df* = 5 Linear trend *p* < 0.001
0 hrs	8.59% (8.13–9.08) ^a^	
0.1–0.9 hrs	8.15% (7.70–8.64) ^b,c,d^	
1–1.9 hrs	8.81% (8.52–9.11) ^e^	
2–2.9 hrs	9.12% (8.86–9.40) ^b^	
3–3.9 hrs	9.07% (8.75–9.40) ^c^	
4– hrs	9.54% (9.18–9.91) ^a,d,e^	
Computer time		4.250; *p* = 0.002; *df* = 4 Linear trend *p* < 0.001
0 hrs	9.29% (9.01–9.58) ^a^	
0.1–0.9 hrs	9.18% (8.89–9.47) ^b^	
1–1.9 hrs	8.96% (8.67–9.25)	
2–2.9 hrs	8.84% (8.47–9.23)	
3– hrs	8.32% (7.90–8.77) ^a,b^	
Sitting elsewhere		0.502; *p* = 0.681; *df* = 3
0 hrs	9.05% (8.84–9.28)	
0.1–0.9 hrs	8.90% (8.53–9.28)	
1–1.9 hrs	8.98% (8.65–9.33)	
2– hrs	9.20% (8.79–9.62)	
Objective sedentary time ^#^		3.22; *p* = 0.041; *df* = 2; Linear trend *p* = 0.016
0–499 min	8.63% (7.94–9.37) ^a^	
499.01–580.9 min	8.96% (8.31–9.67)	
581– min	9.83% (9.16–10.54) ^a^	
Work-related sitting *		0.957; *p* = 0.430; *df* = 4
0 hrs	6.57% (6.30–6.84)	
0.1–0.9 hrs	6.69% (6.32–7.09)	
1–3.9 hrs	6.51% (6.27–6.75)	
4–6.9 hrs	6.41% (6.18–6.65)	
7– hrs	6.33% (6.09–6.57)	

^#^ n = 429; * includes only those currently active in working life n = 3248

Models adjusted for: age, education, employment status (except in model of work-related sitting), study year (except in model with objective sedentary time), BMI, and leisure time physical activity. Statistically significant pairwise comparisons are denoted with the same letter in superscript.

**Table 3.** Framingham risk score in women by type of sitting.

Sedentary measure	Framingham risk score % (Geometric mean and corresponding 95% CI)	Overall F-test, *p*-value, *df*, trend
Total sitting		1.386; *p* = 0.236; *df* = 4 Quadratic trend *p* = 0.034
< 4 hrs	4.22% (4.05–4.40)	
4–5.9 hrs	4.08% (3.90–4.25)	
6–7.9 hrs	4.07% (3.88–4.26)	
8–9.9 hrs	4.13% (3.95–4.33)	
10– hrs	4.20% (4.01–4.39)	
Sitting in vehicle		1.323; *p* = 0.259; *df* = 4
0 hrs	4.07% (3.91–4.23)	
0.1–0.9 hrs	4.21% (4.05–4.38)	
1–1.9 hrs	4.17% (3.99–4.37)	
2–2.9 hrs	4.14% (3.85–4.45)	
3– hrs	4.29% (3.78–4.87)	
TV sitting		4.140; *p* = 0.001; *df* = 5 Linear trend *p* = 0.008
0 hrs	3.98% (3.74–4.23)	
0.1–0.9 hrs	3.91% (3.69–4.15) ^a^	
1–1.9 hrs	4.04% (3.88–4.22) ^b^	
2–2.9 hrs	4.28% (4.11–4.46) ^a,b^	
3–3.9 hrs	4.20% (4.01–4.40)	
4– hrs	4.18% (3.98–4.40)	
Computer time		1.239; *p* = 0.292; *df* = 4
0 hrs	4.21% (4.05–4.39)	
0.1–0.9 hrs	4.13% (3.96–4.30)	
1–1.9 hrs	4.05% (3.87–4.23)	
2–2.9 hrs	4.10% (3.88–4.34)	
3– hrs	4.20% (3.91–4.51)	
Sitting elsewhere		2.183; *p* = 0.088; *df* = 3
0 hrs	4.19% (4.04–4.35)	
0.1–0.9 hrs	4.00% (3.79–4.23)	
1–1.9 hrs	4.09% (3.89–4.31)	
2– hrs	4.05% (3.85–4.25)	
Objective sedentary time ^#^		0.213; *p* = 0.808; *df* = 2
0–499 min	4.10% (3.84–4.38)	
499.01–580.9 min	4.12% (3.87–4.38)	
581– min	3.99% (3.72–4.29)	
Work-related sitting *		0.718; *p* = 0.580; *df* = 4
0 hrs	3.10% (2.94–3.27)	
0.1–0.9 hrs	3.07% (2.91–3.25)	
1–3.9 hrs	3.03% (2.89–3.16)	
4–6.9 hrs	2.98% (2.85–3.12)	
7– hrs	3.03% (2.90–3.17)	

^#^ n = 559; * includes only those currently active in working life n = 3732.

Models adjusted for: age, education, employment status (except in model of work-related sitting), study year (except in model with objective sedentary time), BMI, and leisure time physical activity. Statistically significant pairwise comparisons are denoted with the same letter in superscript.

## 4. Discussion

Our population-based data in 10,185 apparently healthy individuals between the age of 25 and 74-years suggest that out of several type-specific sitting behaviors, only TV sitting showed statistically significant associations with the Framingham score, independent of confounding roles of age, education, employment status, leisure time physical activity, BMI, and study year. In men and women, the lowest risk score was found for sitting in front of the TV from 6 minutes to less than 1 hour daily, while the high risk group in TV sitting was 4 hours or more in men and 2 to 3 hours in women. Of other types of sitting, computer sitting was inversely associated with the Framingham risk in men only. Noteworthy, the total amount of self-reported sitting time had no association with the Framingham score, yet objectively assessed total sedentary time was directly related to the Framingham score in men, with a higher score in the highest third of sitting.

The observed differences in the Framingham score were of modest size and clinical relevance, between one to two percentage units across the sitting groups. It should, however, be born in mind that the analyses were, due to a skewed distribution, carried out using log-transformed Framingham score and the variance of the non-transformed score is substantially higher across categories of sitting. For example, half (52%) of those men with ≥ 4 hours of TV sitting had a Framingham score of higher than 20% (i.e. considered as high risk threshold [31]), as compared to only a tenth (10.9%) of men with TV sitting between 6 minutes and 1 hour. In women, a high Framingham score (> 20%) was present for 20% of women with TV sitting more than 4 hours and for 2.3% of women with TV sitting between 6 minutes and 1 hour.

### 4.1. Comparisons to Previous Findings

While previous studies have reported both direct and non-significant associations between SB and health outcomes, our findings suggest that only TV sitting associates with higher CVD risk estimate, when using the Framingham score. This finding on TV sitting and CVD risk is supported in many cross-sectional and prospective studies [15],[18]–[20],[44]. Similarly to our results on leisure time computer use, previous findings have indicated an inverse cross-sectional association with cardiometabolic risk markers [18] and with obesity [45]. Controversial findings are also reported, with a similar cardiometabolic risk score regardless of time spent in front of a computer [44]. Computer usage is often combined with TV sitting into a single measure of screen time [29],[46]. However, it is reported that the correlates of TV sitting and computer sitting are different [29] and as suggested by our results, the health outcome for these two types of SB may differ.

We acknowledge two previous studies that have reported the association between SB and CVD risk based on a risk index. Fitzgerald et al. [32] reported the association between objectively measured physical activity and SB with the Framingham risk score in a sample of old, mobility limited adults (age 70–89 years). Loprinzi [47] studied the risk of a first atherosclerotic CVD event according to the Pooled Cohort equation in a sample of 40–79 year olds from the NHANES 2003–2006 data. These two previous studies assessed SB objectively and thus discussed only total sedentary time. Both studies concluded on a higher predicted CVD risk with higher SB, although the size of the association was concluded to be small [32],[47]. Other findings from accelerometer-based studies on SB and cardiovascular health have so far been inconclusive. Out of several cardiometabolic risk factors, accelerometer-based SB was only associated with total cholesterol in working-aged adults [48] and waist circumference in older adults [10], while a meta-analysis concluded no significant associations between accelerometer-based SB and blood pressure [49]. The finding among men in our study is supported by that of Fitzgerald et al. [32] and Loprinzi [47], but likewise as these authors, we can conclude that the size of the association is small.

Many studies have found an increased risk of CVD for very high levels of total sitting or TV sitting time when compared to the lowest end of sitting or TV sitting time, but not all observe any significantly increased risk for sitting time in between the two ends [50]. Along these lines, one meta-analysis [16] indicated that the mortality risk associated with every 1-hour increase in sitting time was higher among those with more than 7 hours sitting/day than in those with only 0 to 3 hours sitting/day. For TV sitting, the literature often reports a cutoff at 4 hours for the highest TV time [18],[50]. This was also used as the cutoff for the last group in our analyses and thus makes our result regarding TV sitting comparable to previous findings. Furthermore, as also suggested by others, our pairwise comparisons between the groups of TV sitting against the Framingham score did not suggest all groups to be statistically different from each other. As a consequence, it is only possible to recommend avoidance of very high amounts of TV sitting, as the results do not directly support avoiding all sitting.

Controversially, we observed the highest Framingham score for the group of men that reported no computer time as compared to those reporting some. In contrast to our findings, it was reported in Norwegian adults that recreational computer time was associated with worse CVD risk factor levels [18]. Other studies have also provided mixed findings [27],[28]. However, it is known that younger age relates to more computer time [29] and that higher computer time in young subjects does not seem to be associated with worse cardiometabolic risk profile [44]. This explanation may also be likely in our study.

Occupational sitting has been previously studied in terms of relations to health, although much of the literature has concentrated on the role of physical activity at work, not sitting. We analyzed the work-related sitting only among those currently active in working life. Work-related sitting was not associated with the Framingham score in our analyses. One of the oldest studies originated in the 1950's where physically active occupations protected the heart more than sedentary tasks, yet these findings were partly explained by overweight [51]. Previous studies also have indicated that sitting occupations may be more strongly associated with adverse health among women than men [4],[22],[34], but also that sedentary jobs may be indecisive in the development of incident health problems [15],[52],[53]. The non-significant finding between work-related sitting and the Framingham score may due to a confounding role of socioeconomic status. Highly educated persons are more likely to engage in desk-bound occupations but also in health conscious lifestyles. Employees who sit more during their job are more likely to be physically active in leisure time and to meet the physical activity guidelines, as compared to employees with physically more active jobs [8]. Our findings are in line with the previous evidence indicating that leisure time sitting may be more harmful than occupational sitting for health.

Of other types of SB, information on vehicle sitting and CVD risk is sparse, as most of the evidence had been produced for risk of obesity. Driving a car or time spent in cars has been shown to be associated with a risk of CVD mortality [54], obesity [25],[55] and higher cardiometabolic score [26]. Our findings add to current knowledge by suggesting non-significant associations for vehicle sitting and the Framingham score.

Earlier findings on leisure time sitting (such as TV and/or computer time, reading, or playing games) and health outcomes have also been controversial [15],[27],[48],[56]. Our findings add to current knowledge by suggesting non-significant findings for sitting elsewhere (here referring to other locations but in front of TV or computer or in a vehicle) with CVD risk. Since sitting elsewhere includes very many choices, such as sitting while reading, eating, doing handicrafts, or socializing, it is concluded that our non-significant finding is somewhat expected. The role of social and environmental attributes influencing type-specific SB should be kept in mind particularly when planning interventions and giving advice on SB [57].

In our sample the mean time of self-reported total SB was higher than what has been reported in studies worldwide [58],[59]. In a 20-country comparison the median self-reported daily sitting time was 300 minutes/day, with an interquartile range of 180–480 minutes, among adults aged 18 to 65-years [58]. In the NHANES 2009/2010 sample from the U.S. mean self-reported sitting time was 285 min/day for men and 281 min/day for women [59]. Our measure of self-reported sitting was based on the sum of type-specific sittings and thus it is expected that the amount of total SB can be higher than if total sitting is assessed only by one question as in the compared studies. In our study the mean accelerometer-based SB was somewhat lower than reported in a sample of middle-aged and older U.S. adults (670.2 ± 123.9 min) [60]. Regarding objectively measured SB our sample was younger than the sample from the U.S., probably explaining a great part of the lower objective SB time in our study.

### 4.2. Methodological Considerations

Our study has several methodological considerations that include self-reported data for SB, cross-sectional design, and small sample size for accelerometer-based data. On self-reports, we face the challenge of information bias. The context of sitting is not measurable in any other method but self-report and thus this limitation is a general problem in all large-scale studies. In terms of information bias, we noticed higher Framingham scores in those with no computer sitting. This has not been reported before in other studies, yet one review [16] suggested a non-linear association based on current evidence. It is clear that this issue warrants more research to understand if this is related to issues in self-report techniques or explained by residual confounding. Our study is limited to only cross-sectional associations between SB and the Framingham score. Therefore we are not able to conclude on causality or on which has arrived first — high sitting or high Framingham score. Regarding the generalizability of the sample, it has earlier been shown that non-participation includes more often the lower educated, young men, and old people with poor health status [61],[62]. It is therefore suggested that if the non-participants had been included in the sample, many of the studied associations had been stronger [62]. This assumption is highly likely in our analyses, too. Finally, our study is limited to a small sample size for accelerometer-based SB. It is possible that selection bias is likely in this smaller sample, even it is a part of our representative sample.

This study includes several strengths. These data were collected for monitoring CVD risks factors in the population and thus serve well to study SB. Moreover, data comprised information on underlying CVD conditions that allowed us to include only apparently healthy participants in the analyses. This way we were able to control for reverse causation, where high amounts of sitting may be a result of an existing disease. It should be born in mind that as we only studied cross-sectional associations, reverse causation is an issue that should be taken more seriously into account in SB-related research. Another strength of our study was the large population-based sample that had a high participation rate and is generalizable to the general CVD-free population in Finland, covering broad age groups and both genders.

## 5. Implications

To answer the question “does it matter where to sit?” our data suggest that only sitting in front of TV matters for the risk of future CVD. Furthermore, for TV sitting, the most beneficial Framingham score appeared for those with low-to-moderate amounts of sitting. All other type-specific SBs showed no significant associations with the Framingham score. Our findings implicate that when promoting heart health, the main SB to be targeted is TV viewing. Future research should concentrate on understanding more carefully why TV sitting is more harmful on health than other types of sitting.
